# IAG Regulates the Expression of Cytoskeletal Protein-Encoding Genes in Shrimp Testis

**DOI:** 10.3390/genes14030564

**Published:** 2023-02-23

**Authors:** Qian Lv, Shihao Li, Miao Miao, Songjun Jin, Fuhua Li

**Affiliations:** 1 CAS and Shandong Province Key Laboratory of Experimental Marine Biology, Institute of Oceanology, Chinese Academy of Sciences, Qingdao 266071, China; 2 Laboratory for Marine Biology and Biotechnology, Qingdao National Laboratory for Marine Science and Technology, Qingdao 266237, China; 3 School of Marine Science and Engineering, Qingdao Agricultural University, Qingdao 266109, China; 4 Center for Ocean Mega-Science, Chinese Academy of Sciences, Qingdao 266071, China; 5 University of Chinese Academy of Sciences, Beijing 100049, China; 6 The Innovation of Seed Design, Chinese Academy of Sciences, Wuhan 430072, China

**Keywords:** insulin-like androgenic gland hormone, testis development, transcriptome, cytoskeleton, crustacean

## Abstract

Insulin-like androgenic gland hormone (IAG) is the master regulator of sexual differentiation and testis development in male crustaceans. However, the molecular mechanism on how IAG functions during testis development is still largely unknown. Here, the transcriptional changes were analyzed in the testes of shrimp after *LvIAG* knockdown in *Litopenaeus vannamei*. Differential expression analysis identified 111 differentially expressed genes (DEGs), including 48 upregulated DEGs and 63 downregulated DEGs, in testes of shrimp after *LvIAG* knockdown. Gene ontology (GO) analysis showed that these DEGs were apparently enriched in cytoskeleton-related GO items. Gene function analysis showed that genes enriched in these GO items mainly encoded actin, myosin, and heat shock protein. Interestingly, these genes were all downregulated in testis after *LvIAG* knockdown, which was confirmed by qRT-PCR detection. Furthermore, injection of LvIAG protein that was recombinantly expressed in insect cells upregulated the expression levels of these genes. The present study revealed that shrimp IAG might function in testis development through regulating the expression of cytoskeletal protein-encoding genes, which would provide new insights into understanding the functional mechanisms of IAG on male sexual development of crustaceans.

## 1. Introduction

The sexual development process of male crustaceans is widely believed to be under regulation of the “eyestalk–androgenic gland (AG)–testis” endocrine axis [1]. The insulin-like androgenic gland hormone (IAG), secreted by AG, works as the master regulator of sexual differentiation and testis development in male crustaceans. The IAG, previously called androgen gland hormone (AGH), was firstly isolated from the androgenic gland of the isopod *Armadillidium vulgare* [2]. It has a structure similar to insulin, consisting of a signal peptide at the N-terminal, a B chain, a C peptide, and an A chain, and generates the mature peptide by removing the C peptide and forming two disulfide bonds between the B chain and the A chain [3,4]. 

IAG is considered as the sexual “IAG-switch” owing to its roles during sexual development in male crustaceans [5]. In *Lysmata wurdemanni*, IAG is considered to play a role in controlling male differentiation in dioecious, sequential, and prohermaphrodite decapod crustacean sexual systems [6]. In the male *Macrobrachium rosenbergii*, gonad differentiation, secondary sex trait development, and spermatogenesis could be inhibited, and the functional sex-reversal female could be generated after silencing the expression of the *MrIAG* gene using RNA interference (RNAi) [7,8,9]. Transplantation of the androgenic gland from *Procambarus clarkii* into the female *Procambarus fallax f. virginalis* caused masculinization of several secondary sex characteristics in the later crayfish [10]. In the precocious *Eriocheir sinensis*, IAG plays a vital function in maintaining male characteristics and promoting testicular development [11]. These studies provide solid evidence for the effect of IAG on sexual differentiation and development in male crustaceans.

Although IAG hormone functions on sexual differentiation and gonad development of male crustaceans, the underlying molecular mechanisms are still less studied. In some crustaceans, several IAG-binding proteins or putative receptors have been reported. In the crayfish *Cherax quadricarinatus*, an insulin-like growth factor-binding protein was found interacting with the IAG hormone [12]. In *M. rosenbergii* and *Macrobrachium nipponense*, the expression of IAG shows a regulatory relationship with the insulin-like androgenic gland hormone-binding protein [13,14]. Several tyrosine kinase receptors were identified as the putative IAG receptors and played important functions in male sexual development. In *Fenneropenaeus chinensis*, an IAG receptor (FcIAGR), mainly expressed in the androgenic gland and testis, and functional analysis showed that the development of germ cells in testis could be arrested after knockdown of *FcIAGR* [15]. In *M. rosenbergii*, knockdown of an insulin-like receptor (MrIR) generates the sex-reversal individuals, suggesting that MrIR might be a receptor for IAG hormone [16]. In *Sagmariasus verreauxi*, the tyrosine kinase insulin receptor (Sv-TKIR) is mainly expressed in gonad and antennal glands of male individuals [17]. Furthermore, the secretory production of AG activates protein kinases (PKs) or phosphatases (PPs) in *P. clarkii*, directly enhancing phosphorylation of some testicular peptides and decreasing phosphorylation of some other proteins, which indicated that the IAG receptor in testis recognized the AG production and then regulated cell proliferation and differentiation of spermatids [1,18,19]. These findings partially reveal how IAG functions during sexual development in male crustaceans. However, more studies are needed to illustrate the functioning mechanisms of IAG on testis development.

Considering that IAG has diverse functions in male development, including gonad differentiation, spermatogenesis, and sexual development of the secondary characteristics, the present study focused on the process of spermatogenesis in adult shrimp. Here, a comparative transcriptomic analysis was performed on testis before and after *LvIAG* knockdown from the shrimp *Litopenaeus vannamei*. The identified differentially expressed genes were mainly clustered into cytoskeleton and related gene ontology (GO) items. Most of these genes were downregulated in the testis after *LvIAG* knockdown, while they were upregulated after injection of recombinant LvIAG protein. The present data provided new understandings in revealing the functional mechanisms of IAG on male sexual development in crustaceans.

## 2. Materials and Methods

### 2.1. Animals

The shrimp with a body length of 12 ± 0.2 cm and a body weight of 7 ± 0.8 g were purchased from a culturing farm near Qingdao and temporarily raised in the aquarium in our laboratory. The temperature of the aerated seawater was controlled at 25–26 °C. The photoperiod during the husbandry condition was 10L:14D. The shrimp were fed twice with an artificial diet (Dale, Yantai, China) and the water was changed by 70% daily. 

### 2.2. dsRNA Preparation

The cDNA template for dsRNA synthesis of *LvIAG* (NCBI accession: XM_027374208.1) was amplified by primers *LvIAG*-dsF/dsR (Table S1). The DNA fragments of the enhanced green fluorescent protein (EGFP) gene were amplified by primers of EGFP-dsF/dsR (Table S1) and used as the template for synthesis of the negative control dsRNA. The PCR was carried out as follows: denaturation at 94 °C for 4 min, 35 cycles of denaturation at 94 °C for 30 s, annealing at 55 °C for 30 s, and extension at 72 °C for 30 s, followed by an extension at 72 °C for 10 min. The amplified DNA product was purified using the Steady Pure PCR DNA Purification Kit (agbio, Hunan, China) and evaluated by 1.5% agarose gel electrophoresis. The dsRNA was synthesized with 1 µg template DNA using the Transcript Aid T7 High-Yield Transcription Kit (Thermo Fisher Scientific, Waltham, MA, USA). After RNaseA (Thermo Fisher Scientific, Waltham, MA, USA) digesting, synthesized dsRNAs (dsLvIAG and dsEGFP) were assessed by 1.5% agarose gel electrophoresis and quantified with the Nanodrop2000 (Thermo Fisher Scientific, Waltham, MA, USA).

### 2.3. RNA Interference and Tissue Collection

The male shrimp in the inter-molt stage were separated into two groups with 22 individuals in each group and injected with dsLvIAG and dsEGFP intramuscularly at the fourth abdominal segment, respectively. Preliminarily, 2 μg, 4 μg, and 8 μg of dsRNA was injected into each shrimp to optimize the dosage, and 8 μg of dsRNA per shrimp showed effective RNA interference efficiency. Therefore, we used 8 μg of dsRNA per shrimp for the RNA interference experiment. The shrimp were injected with the same dose of dsEGFP for the control group. Testis was dissected individually for expression analysis of *LvIAG* at 48 h post-dsRNA injection. The collected samples were pre-treated in liquid nitrogen and then stored at −80 °C before use.

### 2.4. Extraction of Total RNA and Synthesis of cDNA

Total RNA was isolated from these samples using RNAiso Plus reagent (TaKaRa, Kyoto, Japan) following the manufacturer’s instructions. The total RNA was assessed by 1% agarose gel electrophoresis and Nanodrop2000 (Thermo Fisher Scientific, Waltham, MA, USA). The genomic DNA was removed by DNase I and 1 µg of total RNA was used to synthesize the cDNA using the PrimeScript RT Reagent Kit (TaKaRa, Kyoto, Japan).

### 2.5. Quantitative Real-Time PCR (qRT-PCR)

The relative expression level of *LvIAG* in testis after gene knockdown was detected by qRT-PCR. The 18S rRNA was used as an internal reference gene. The primers *LvIAG*-qF/qR (Table S1) were used following the program: denaturation at 94 °C for 5 min, followed by 40 cycles of 95 °C for 15 s, 56 °C for 20 s, and 72 °C for 30 s. The 10 μL RT-PCR reaction mixture consisted of 5 μL of SYBR qPCR Mix (Toyobo, Osaka, Japan), 1 μL of diluted cDNA, 0.3 μL of forward/reverse primer, and 3.4 μL of RNase-free waters. The specificity of amplification was evaluated according to the melting curve. Four technical replications were set for each sample. The relative expression level of the *LvIAG* was obtained by the 2^–ΔΔCT^ method [20]. The statistically significant difference between the two groups was analyzed by the independent sample *t*-test using GraphPad Prism software (version 7.0) at a *p*-value < 0.05.

### 2.6. Illumina Sequencing and De Novo Assembly 

Nine samples were selected in the dsLvIAG and dsEGFP groups, respectively, according to the expression levels of *LvIAG*. Equal quantities of total RNA were obtained from three samples and mixed as one biological replicate in each group. Finally, three biological replicates were obtained for each group. These samples were designated as IAT1, IAT2, and IAT3 in the dsLvIAG group, and ICT1, ICT2, and ICT3 in the dsEGFP group. The mRNA was isolated by magnetic beads with Oligo(dT) and interrupted under ultrasound. Fragment mRNA was used to obtain the first strand of cDNA by the M-MLV reverse transcriptase with random oligonucleotides as primers. Then, the RNA strand was degraded by RNase H, and the second strand of cDNA was obtained using DNA Polymerase I. The purified cDNA was tailed by adding A and connected with sequencing adaptors. The cDNA with a length of about 200 bp was screened with agarose gel electrophoresis, amplified by PCR, and then purified for library construction and sequencing by Illumina HiSeq 2500 at Gene Denovo Biotechnology Co. (Guangzhou, China). Prior to mapping and assembly, raw reads were removed, and only reads with a mass fraction above 10 were utilized for further analysis.

### 2.7. Identification, Annotation, and Validation of Differentially Expressed Genes (DEGs)

The reads were assembled and the fragments per kilobase of transcript sequence per million base pairs sequenced (FPKM) of the identified genes were computed [21]. The *p*-values were adjusted by the false detection rate (FDR) method [22]. The criteria for DEGs were set to fold change of expression level ≥2 and FDR < 0.05. Then, the DGEs were enriched by Gene Ontology (GO) terms and Kyoto Encyclopedia of Genes and Genomes (KEGG) pathways. Seven differentially expressed genes (DEGs) were randomly selected to verify the transcriptome data using the qRT-PCR method, as described in Section 2.5. The primers are listed in Table S1.

### 2.8. Gene Cloning and Construction of the Recombinant Bacmid

The primers *LvIAG*-F/R (Table S1) were used to amplify the full length of the *LvIAG* coding region. The cDNA fragments were amplified using the PrimeSTAR GXL DNA Polymerase kit (TaKaRa, Kyoto, Japan) as follows: denaturation at 98 °C for 5 min, followed by 40 cycles of denaturation at 98 °C for 10 s, annealing at 55 °C for 10 s, and extension at 68 °C for 10 s, and then extended at 68 °C for 10 min. The PCR products were detected by 1% agarose gel electrophoresis and purified using the Steady Pure PCR DNA Purification Kit (Agbio, Hunan, China). PCR products were linked to the pMD19-T vector (TaKaRa, Kyoto, Japan) for 4 h at 16 °C, and transformed into Trans5α competent cells (TransGen Biotech, Beijing, China). The single colony was selected for PCR detection by 1% agarose gel electrophoresis. The positive clones with the expected size were selected and sent for sequencing. 

The pFastBac1 plasmid (Invitrogen, CA, USA), which was modified by adding the His-tag and FLAG-tag before multiple cloning sites, was digested by *EcoR* I and *Sal* I to generate a linearized vector. The PCR products were amplified using the primer *LvIAG*-pFastBac F/R (Table S1) and subcloned into the linearized pFastBac1 plasmid by the Infusion HD Cloning Kit (Clontech, Mountain View, CA, USA). The plasmid, named pFastBac-IAG, was extracted, verified, and transformed into DH10Bac^TM^ competent cells (AngYuBio, Shanghai, China). Then, it was cultured on the LB plate containing tetracycline (10 μg/mL), kanamycin sulfate (50 μg/mL), gentamicin (7 μg/mL), IPTG (40 μg/mL), and X-Gal (40 μg/mL) at 37 °C for 48 h to select the positive clone. The white spot monoclonal was transferred into the LB fluid containing tetracycline (10 μg/mL), kanamycin sulfate (50 μg/mL), and gentamicin (7 μg/mL) and cultured at 225 r/min, 37 °C, for 12 h. The Bacmid, named *LvIAG*-Bacmid, was extracted with the Invitrogen™ Pure Link™ HiPure Plasmid Microextraction Kit (Thermo Fisher Scientific, MA, USA) and stored at −20 °C.

### 2.9. Expression and Purification of Recombinant Protein

ExpiSf9™ cells were sub-cultured and expanded until the cells reached a density of approximately 5~10 × 10^6^ cells/mL. The *LvIAG*-Bacmid was transferred into ExpiSf9™ cells and viruses were collected after the cells showed pathological changes. Freshly cultured ExpiSf9™ cells were infected with the viruses and then collected when they showed pathological changes at about 120 h post-infection, and the collected cells were lysed by adding xTractor buffer (TaKaRa, Kyoto, Japan) and Halt Protease Inhibitor Cocktail, EDTA-Free (Thermo Fisher Scientific, MA, USA). According to the specification of xTractor Buffer, 20 mL was used per 1 g of cells, and immediately before use, 10 µL of Halt Protease Inhibitor Cocktail per milliliter of sample was directly added to the lysis buffer. The supernatant was collected after centrifugation of cell lysate at 4 °C, 12,000× *g* for 15 min. The recombinant LvIAG protein was purified by the HisTALON™ Gravity Column Purification Kit (Clontech, CA, USA) and verified by SDS-PAGE electrophoresis and Western blot. The Western blot was performed using the primary anti-Flag antibody (Abcam, Cambridge, UK) and the anti-rabbit IgG (Cell Signaling Technology, MA, USA). The recombinant protein was dialyzed using PBS and quantified with the BCA Protein Quantification Kit (Vazyme, Nanjing, China).

### 2.10. Recombinant Protein Injection and Sampling

Four groups were set, with five male shrimp in each one. The first group was injected with 6 μg of recombinant LvIAG protein and the second group was injected with 10 μg of recombinant LvIAG protein for each shrimp. The control group was injected with PBS. Shrimp testis was dissected at 24 h post-protein injection. Total RNA extraction, cDNA synthesis, and qRT-PCR analysis of selected DEGs were performed following the methods in Section 2.4 and Section 2.5. 

### 2.11. Statistical Analysis

The qPCR data were presented in the form of the mean ± S.E. The independent *t*-test was performed to calculate the significant difference between two treatments by SPSS 25.0 software (https://www.ibm.com/spss, accessed on 18 April 2022). Significant differences between two treatments were shown at *p* < 0.05 (*) and *p* < 0.001 (**).

## 3. Results

### 3.1. The Basic Information of the Transcriptome 

**Figure 1:**
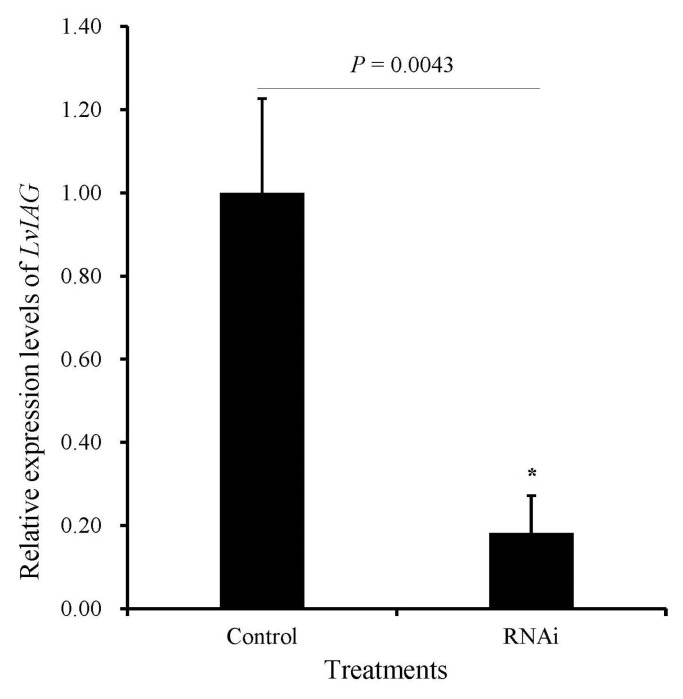
Expression analysis of *LvIAG* in the androgenic gland of shrimp before and after RNA interference. Significant downregulation of *LvIAG* was detected after RNA interference. RNAi showed the group after *LvIAG* knockdown, and control showed the group before *LvIAG* knockdown. The significant difference was shown with (*) at *p <* 0.05.

**Figure 2:**
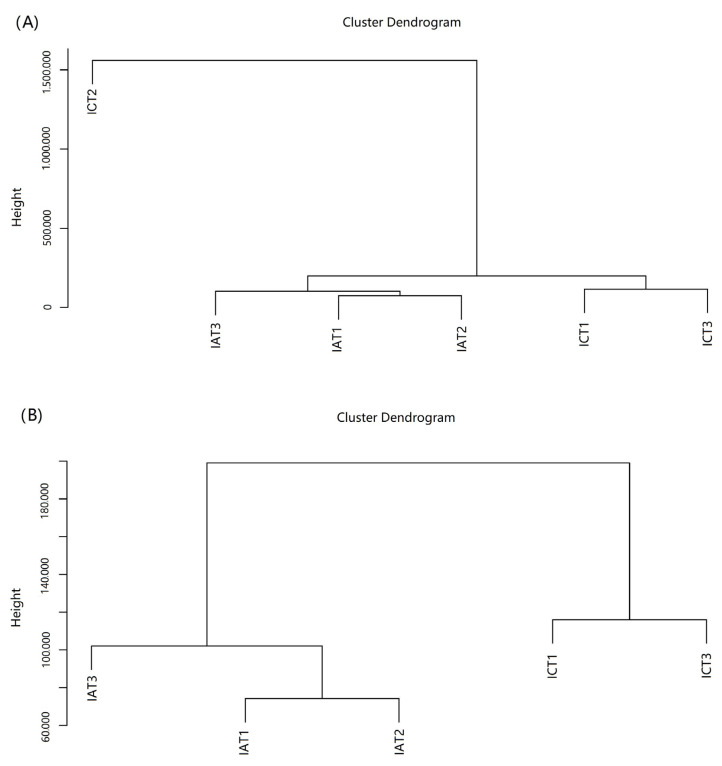
Hierarchical clustering analysis (HCA) of all gene expression profiles of samples from two treatments. The deviation of ICT2 was too large and was removed in further analysis. (**A**) The HCA results of all six samples. (**B**) The HCA results after removing the sample ICTs. IAT1, IAT2, and IAT3 are three replicates in the dsLvIAG group, and ICT1, ICT2, and ICT3 are three replicates in the dsEGFP group.

Expression analysis revealed that the relative expression level of *LvIAG* in the androgenic gland of shrimp from the dsLvIAG group was significantly reduced by 82% compared with that from the dsEGFP group (Figure 1). Therefore, testes from these individuals were collected and used for the subsequent comparative transcriptome analysis. Each group set three biological replicates. After gene assembly, hierarchical clustering analysis (HCA) of these samples showed that the deviation of ICT2 was too large (Figure 2A). Therefore, ICT2 was deleted in the subsequent transcriptome analysis (Figure 2B). 

### 3.2. Differentially Expressed Genes (DEGs) between Two Treatments

**Figure 3:**
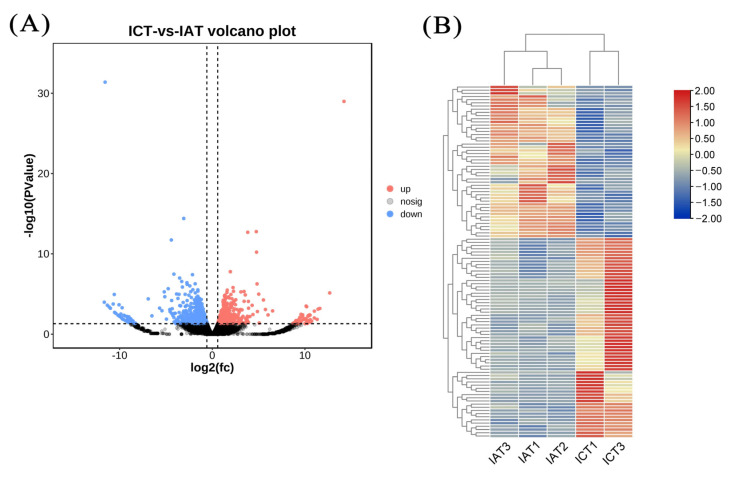
Identification of DEGs between two treatments. Volcano plot analysis (**A**) and heatmap analysis (**B**) of the DEGs were displayed. A total of 111 DEGs, including 63 downregulated DEGs and 48 upregulated DEGs, were identified in the RNAi group.

Differential expression analysis identified 111 DEGs between two treatments, including 63 downregulated DEGs and 48 upregulated DEGs in the RNAi group (Figure 3). The annotation details of all DEGs are listed in Table S2, and there were 82 DEGs with functional annotations.

**Figure 4:**
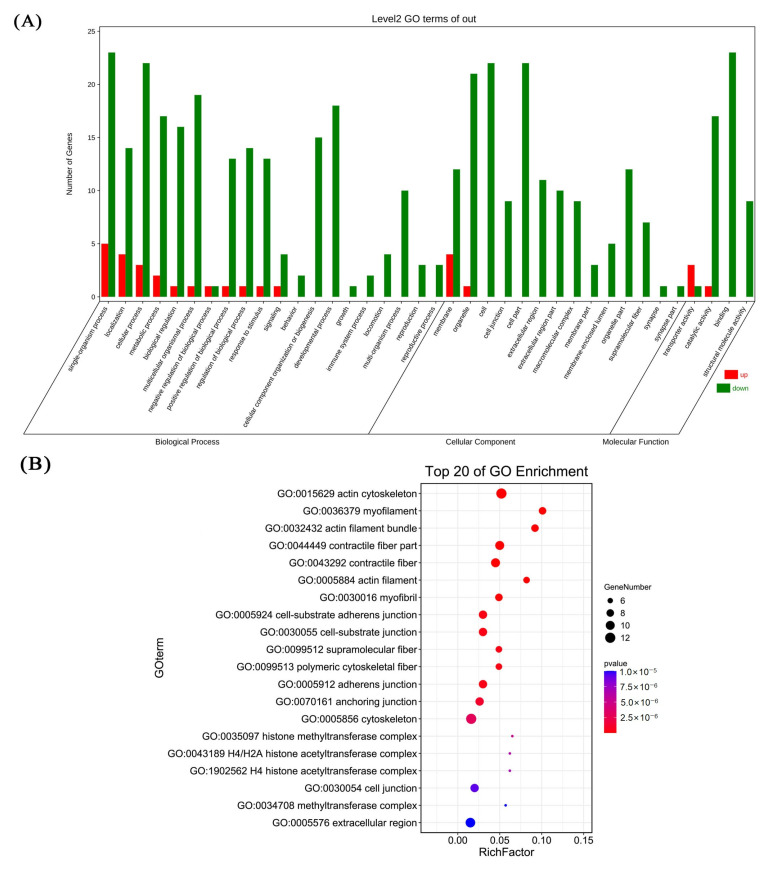
Gene ontology (GO) enrichment (**A**) and the top 20 cell components of GO enrichment (**B**) of DEGs from the transcriptome data. DEGs were enriched in 38 GO items. Many items related to the cytoskeleton were enriched in the top 20 cell components. Up and down in (**A**) represent upregulated and downregulated DEGs in the *LvIAG* knockdown treatment.

The biological functions of 111 DEGs were further studied by Gene Ontology (GO) enrichment and the Kyoto Encyclopedia of Genes and Genomes (KEGG) pathway analyses. In total, 111 DEGs were enriched in 38 GO items, among which 20 were enriched in biological processes, four in molecular functions, and 14 in cellular components (Figure 4A). In “biological process”, DEGs were mainly enriched in the single-organism process, cellular process, and multicellular organismal process. In “cell component”, DEGs were mainly enriched in the cell part and the organelle. In “molecular function”, DEGs were mainly enriched in binding, the multicellular organismal process, the developmental process, and other categories.

In the top 20 cell components of GO enrichment, many items related to the cytoskeleton, including the actin cytoskeleton, myofilament, actin filament bundle, contractile fiber part, contractile fiber, actin filament, myofibril, and so on, were enriched (Figure 4B). KEGG analysis showed that only two pathways, including glycosaminoglycan biosynthesis-keratan sulfate and spinocerebellar ataxia, were enriched with one gene in each pathway at *p*-value ≤ 0.05.

### 3.3. Cytoskeleton Related Genes Were Downregulated in Shrimp Testis after LvIAG Knockdown

**Figure 5:**
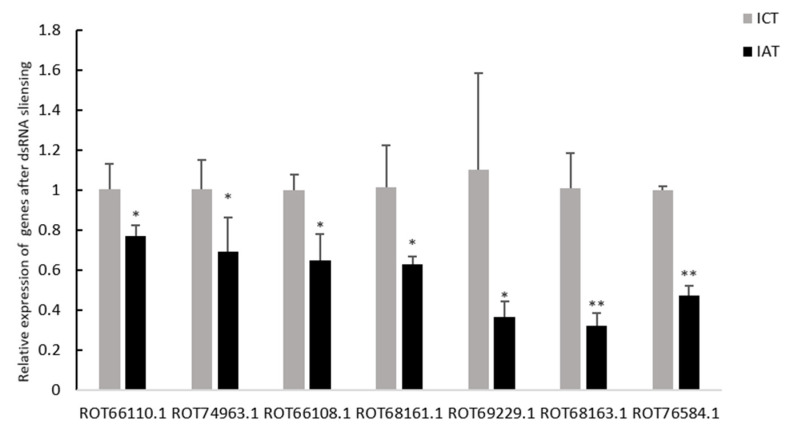
The expression analysis of seven selected DEGs by qRT-PCR in shrimp before and after *LvIAG* knockdown. ROT66110.1, ROT74963.1, ROT66108.1, and ROT69229.1 encode actin 2. ROT68161.1 and ROT68163.1 encode myosin heavy chain type 2. ROT76584.1 encodes myosin light chain 2. These DEGs were significantly downregulated in shrimp after *LvIAG* knockdown. IAT: the group after *LvIAG* knockdown. ICT: the control group. The data were presented as mean ± SEM of three biological replicates. Significant differences between two treatments were shown at *p* < 0.05 (*) and *p* < 0.001 (**).

To further study the biological functions of the identified DEGs, genes enriched in the top 20 cell components of GO enrichment were specifically analyzed. DEGs enriched in these items were mainly annotated as heat shock protein, actin, myosin, and kinesin-like calmodulin-binding protein (Table 1). Notably, these DEGs were all downregulated after *LvIAG* knockdown. To further verify the accuracy of the transcriptome data, seven DEGs were randomly selected for qRT-PCR analysis. The results showed that all the selected DEGs were significantly downregulated in shrimp after *LvIAG* knockdown (Figure 5), which was consistent with their expression trends in the transcriptome data.

### 3.4. Cytoskeleton-Related Genes Were Upregulated in Shrimp Testis after Injection with Recombinant LvIAG Protein

**Figure 6:**
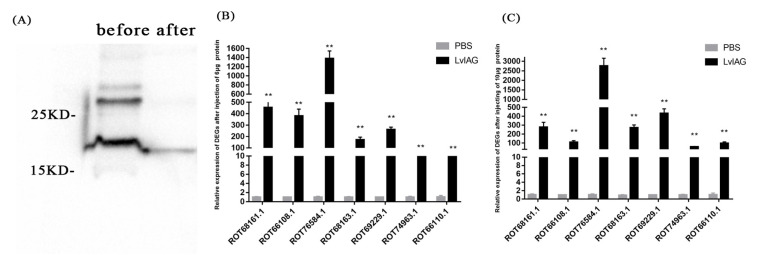
The expression analysis of seven selected DEGs by qRT-PCR in shrimp before and after recombinant LvIAG injection. ROT66110.1, ROT74963.1, ROT66108.1, and ROT69229.1 encode actin 2. ROT68161.1 and ROT68163.1 encode myosin heavy chain type 2. ROT76584.1 encodes myosin light chain 2. The expression levels of these DEGs showed a trend of upregulation after recombinant LvIAG injection. (**A**) Western blot assay of the recombinant LvIAG protein. Before: the recombinant LvIAG protein before purification, and after: the recombinant LvIAG protein after purification. (**B**) The expression level of selected DEGs after injecting 6 μg of recombinant LvIAG protein. (**C**) The expression level of selected DEGs after injecting 10 μg of recombinant LvIAG protein. Significant differences between two treatments were shown at *p* < 0.001 (**).

The Western blot assay was performed to validate the recombinant LvIAG protein. The result showed that both the pre-purified and purified protein had clear bands between 15 and 25 kD in size (Figure 6A), consistent with the predicted size of recombinant LvIAG protein. 

After injection of two recombinant IAG proteins into the shrimp, the expression levels of the differentially expressed genes showed a trend of upregulation to varying degrees. The expression levels of ROT74963.1 and ROT66110.1 were upregulated by 31- and 41-fold compared to control after injection of 6 μg of the recombinant LvIAG protein. The expression levels of ROT69299.1, ROT68163.1, ROT68161.1, and ROT66108.1 were uniformly upregulated by more than 170-fold compared to control. The expression level of ROT76584.1 was upregulated by 1382-fold (Figure 6B). After injecting 10 μg of recombinant protein, the expression of most DEGs showed higher levels while the upregulated expression level of ROT66108.1 decreased compared to those in shrimp after injection with 6 μg recombinant protein (Figure 6C). These results proved that overexpression of the *LvIAG* gene upregulated the expression of microtubules, microfilaments, and skeleton protein-encoding genes in the testis of shrimp. 

## 4. Discussion

Spermatogenesis is a highly complex physiological process in which sperm cells undergo a series of complicated differentiation and morphological changes to form zygotes, including nuclear remodeling, acrosome formation, and caudal formation [23]. The process is coordinated by diverse cytokines and signaling pathways. Cytoskeletal proteins play a variety of roles in determining cell shape, cell motility, maintaining cell connections, and intracellular transport. These biological functions all contribute to maintaining normal function and morphology of epithelia [24]. In eukaryotic cells, the cytoskeleton is comprised of actin, microtubule (MT), and intermediate filament (IF) networks. The MT-based cytoskeleton works in conjunction with the actin-based cytoskeleton to provide a structural basis for intracellular organelles, e.g., endosome-based vesicles and phagosomes, which function to maintain spermatogenic epithelial homeostasis [25]. In addition, MTs also act as a tract to support and facilitate germ cell transport [26]. 

In the present study, although DEGs in shrimp testis after *LvIAG* knockdown were enriched in a number of GO items, many of the GO items were related to the cytoskeleton. Annotation of the genes enriched in these GO items also supported the opinion that knockdown of *LvIAG* affected the expression of many cytoskeleton-related protein-encoding genes. The data indicated that the expression of cytoskeleton-related proteins might be regulated by *LvIAG*. Actin and myosin are two vital kinds of cytoskeleton proteins during spermatogenesis [27]. β-actin, which widely exists in the pachytene spermatocytes, probably participates in pairing of homologous chromosome and synaptonemal complex formation during meiosis, and plays an important function in lengthening the sperm nuclei [28]. The interaction of actin and microtubule can modulate the cell nucleus anchoring during spindle assembly and rotation in meiosis [29]. Myosin Va, a component of the acroplaxome, could help in the fusion of proacrosomal vesicles into acrosome and link acrosome to acroplaxome, which is vital in proacrosomal vesicle transport [26,30]. In four decapod crabs, including *Clibanarius erythropus*, *Maja squinado*, *Cancer pagurus*, and *Potamon fluviatile*, the cytoskeleton protein actin widely exists in the acrosome vesicle and perforatorial column, indicating its function in the acrosome reaction and subsequent fertilization events [31]. The kinesin KIFC1 was involved in acrosome formation and mainly related to a new cytoskeletal structure called acroframosome during spermiogenesis in the caridean shrimp *Exopalaemon modestus* [32]. Knockdown of *LvIAG* downregulated the expression of several genes encoding actin and myosin proteins, while overexpression of *LvIAG* dramatically upregulated their expression levels, suggesting that the cytoskeleton-related genes might be positively regulated by *LvIAG*. In view of the essential function of IAG during male sexual development, we considered that IAG might regulate testis development, i.e., spermatogenesis in crustaceans through affecting the function of the cytoskeleton proteins actin and myosin.

Heat shock proteins are molecular chaperones involved in protein folding, assembly, and transportation, and play important roles in regulating cell growth, survival, differentiation, and spermatogenesis [33,34]. In mice, loss of function of the HSP70 disrupted synaptonemal complex desynapsis and meiosis of spermatocytes [35]. In the crustaceans *Eriocheir sinensis* and *Penaeus monodon*, HSP70 is highly expressed in spermatogenic cells of normal testicular tissue and might play an important role in spermatogenesis [36,37]. In addition, some small HSPs directly interact with cytoskeleton proteins and jointly function in many physiological processes [38]. The expression levels of two genes encoding heat shock proteins were also significantly reduced after *LvIAG* knockdown, indicating that they might be related to the meiosis of spermatogonia cells in shrimp testis in collaboration with cytoskeleton proteins. 

## 5. Conclusions

As the key regulator of male sexual development, IAG regulates testis development, i.e., spermatogenesis in crustaceans. The present study identified the possible target proteins in shrimp testis through transcriptome analysis and subsequent experimental verification. DEGs in testis after *LvIAG* knockdown were mainly enriched in cytoskeleton-related GO items, and these genes were positively regulated by *LvIAG*. The data suggest that *LvIAG* might regulate testis development in adult shrimp through affecting the function of the cytoskeleton. The study provides new insights into the regulatory mechanism of IAG on the sexual development of male crustaceans.

## Figures and Tables

**Table 1:** The expression levels and annotation of DEGs in the top 20 enriched GO items.

Gene ID	ICT Mean	IAT Mean	log2(fc)	*p*-Value	FDR	Annotation
ROT62016.1	0.98	0.03	−5.13	2.19 × 10^−5^	1.10 × 10^−2^	heat shock protein 70 kDa, partial (*Cyanagraea praedator*)
ROT66108.1	58.26	19.76	−1.56	3.03 × 10^−6^	3.10 × 10^−3^	actin 2 (*Penaeus monodon*)
ROT66110.1	10.59	0.73	−3.86	1.01 × 10^−5^	6.80 × 10^−3^	actin 2 (*Penaeus monodon*)
ROT68161.1	29.37	8.92	−1.72	1.34 × 10^−4^	3.38 × 10^−2^	myosin heavy chain type 2 (*Litopenaeus vannamei*)
ROT68163.1	22.98	7.08	−1.70	2.29 × 10^−4^	4.67 × 10^−2^	myosin heavy chain type 2 (*Litopenaeus vannamei*)
ROT69229.1	95.13	26.77	−1.83	7.19 × 10^−6^	5.15 × 10^−3^	actin 2 (*Penaeus monodon*)
ROT74963.1	37.15	8.45	−2.14	3.87 × 10^−8^	1.00 × 10^−4^	actin 2 (*Penaeus monodon*)
ROT75659.1	7.09	1.32	−2.42	9.74 × 10^−7^	1.29 × 10^−3^	actin 2 (*Penaeus monodon*)
ROT76584.1	289.77	88.38	−1.71	4.24 × 10^−5^	1.67 × 10^−2^	myosin light chain 2 (*Procambarus clarkii*)
ROT78241.1	190.02	44.36	−2.10	2.93 × 10^−6^	3.10 × 10^−3^	actin 1 (*Fenneropenaeus chinensis*)
ROT78243.1	20.32	3.05	−2.74	3.90 × 10^−7^	6.77 × 10^−4^	actin 1 (*Fenneropenaeus chinensis*)
ROT78244.1	9.00	0.92	−3.30	2.71 × 10^−7^	5.10 × 10^−4^	actin 1 (*Fenneropenaeus chinensis*)
ROT81749.1	2.20	0.33	−2.72	6.61 × 10^−5^	2.13 × 10^−2^	heat shock protein (*Cherax destructor*)

## Data Availability

The original contributions presented in the study are included in the article/Supplementary Material. Further inquiries can be directed to the corresponding authors.

## Supplementary Materials

The following supporting information can be downloaded at: https://www.mdpi.com/article/10.3390/genes14030564/s1, Table S1: Primer sequence information used in the present study. Table S2: Differentially expressed genes identified from the transcriptome data.
