# Supplementary material for: IAG Regulates the Expression of Cytoskeletal Protein-Encoding Genes in Shrimp Testis
Source: Genes (Basel). 2023 Feb 23;14(3):564. doi: 10.3390/genes14030564 (PMC10048434; doi:10.3390/genes14030564)
Supplement: Supplementary file 1 [file genes-14-00564-s001.zip › Table S1 Primer sequence information used in the present study.pdf]

Table S1 Primer sequence information used in the present study

Primer Name	Nucleotide Sequences (5′-3′)	Annealing Temperature (°C)
Lv IAG-qF	AGTGTCAAGGTCAGCCGATAC	54
Lv IAG-qR	CTGAATCCAACGTGGAATCTCG	
Lv IAG-dsF	TAATACGACTCACTATAGGGTTACTCGGATTGCTGATGCT	60
Lv IAG-dsR	GGGTAGCGGCTGAAGCACTGCCCTATAGTGAGTCGTATTA	
EGFP-dsF	TAATACGACTCACTATAGGGCAGTGCTTCAGCCGCTACCC	62
EGFP-dsR	AAGAACGGCATCAAGGTGAACTCCCTATAGTGAGTCGTATTA	
Lv IAG-F	CTCCAGTTCCCTTCCATCCA	68
Lv IAG-R	CCATAGGTCAAGGCAGGTTC	
p Fast Bac-IAG-F	GAAGCGCGCGGAATTTACAACGTCACGG	55
p Fast Bac-IAG-R	AGGACCCGTACTTCTAAGTCGACGAGCTCACTA	
ROT68161.1-F	TAAGGGACGCATCATTTC	60
ROT68161.1-R	GCCACTGAACTTGATGCT	
ROT66108.1-F	TTACCCACTACCTGATGAAGA	60
ROT66108.1-R	CTTTGCTACATCGCCCTT	
ROT76584.1-F	CCCCATCAACTTCACCACC	60
ROT76584.1-R	GATTGAGGCAGAGCAGTTCC	
ROT68163.1-F	TAAGGGACGCATCATTTC	60
ROT68163.1-R	GCCACTGAACTTGATGCT	
ROT69229.1-F	AACTGGGATGACATGGAGA	60
ROT69229.1-R	GCCACCTATGTTACCATCC	
ROT74963.1-F	GCCGTGCTGTCCCTCTAT	60
ROT74963.1-R	TGTTTGAAGGTTTCGCCC	
ROT66110.1-F	GCCGTGCTGTCCCTCTAT	60
ROT66110.1-R	TGTTTGAAGGTTTCGCCC	
